# Neonatal Seizure Detection Using a Wearable Multi-Sensor System

**DOI:** 10.3390/bioengineering10060658

**Published:** 2023-05-29

**Authors:** Hongyu Chen, Zaihao Wang, Chunmei Lu, Feng Shu, Chen Chen, Laishuan Wang, Wei Chen

**Affiliations:** 1 Greater Bay Area Institute of Precision Medicine, Guangzhou 511466, China; 2 School of Information Science and Technology, Fudan University, Shanghai 200438, China; 3 National Health Commission Key Laboratory of Neonatal Diseases, Department of Neonatology, Children’s Hospital of Fudan University, Shanghai 200433, China; 4 Collaborative Innovation Center of Polymers and Polymer Composites, Department of Macromolecular Science, Fudan University, Shanghai 201203, China

**Keywords:** wearable sensor, neonatal seizure, muti-sensor platform, seizure detection

## Abstract

Neonatal seizure is an important clinical symptom of brain dysfunction, which is more common in infancy than in childhood. At present, video electroencephalogram (VEEG) technology is widely used in clinical practice. However, video electroencephalogram technology has several disadvantages. For example, the wires connecting the medical instruments may interfere with the infant’s movement and the gel patch electrode or disk electrode commonly used to monitor EEG may cause skin allergies or even tears. For the above reasons, we developed a wearable multi-sensor platform for newborns to collect physiological and movement signals. In this study, we designed a second-generation multi-sensor platform and developed an automatic detection algorithm for neonatal seizures based on ECG, respiration and acceleration. Data for 38 neonates were recorded at the Children’s Hospital of Fudan University in Shanghai. The total recording time was approximately 300 h. Four of the patients had seizures during data collection. The total recording time for the four patients was approximately 34 h, with 30 seizure episodes recorded. These data were evaluated by the algorithm. To evaluate the effectiveness of combining ECG, respiration and movement, we compared the performance of three types of seizure detectors. The first detector included features from ECG, respiration and acceleration records; the second detector incorporated features based on respiratory movement from respiration and acceleration records; and the third detector used only ECG-based features from ECG records. Our study illustrated that, compared with the detector utilizing individual modal features, multi-modal feature detectors could achieve favorable overall performance, reduce false alarm rates and give higher F-measures.

## 1. Introduction

Neonatal seizure is an important clinical symptom of brain dysfunction, which is more common in infancy than in childhood [1]. Seizures are usually manifested by behavioral and physiological signal changes, such as repeated movements of the arms, hands, legs and eyes, and are sometimes accompanied by muscle contraction at a fluctuant velocity in the opposite direction [1], as well as changes in the patterns of electroencephalogram (EEG), electrocardiogram (ECG) and respiration, etc. It is reported that the overall seizure morbidity is approximately 1-5% in neonates and 6-13% in preterm infants, which is higher than in normal newborns [1,2]. Compared with children and adults, newborns are more likely to have convulsion persistence or electric seizure persistence. Although the immature brain has a relatively stronger tolerance to convulsions, clinical follow-up and magnetic resonance spectroscopy (MRS) have proved that frequent and continuous convulsions have more serious deleterious effects on brain development and may even cause varying degrees of brain injury and neurological sequelae [3,4].

**Figure 1:**
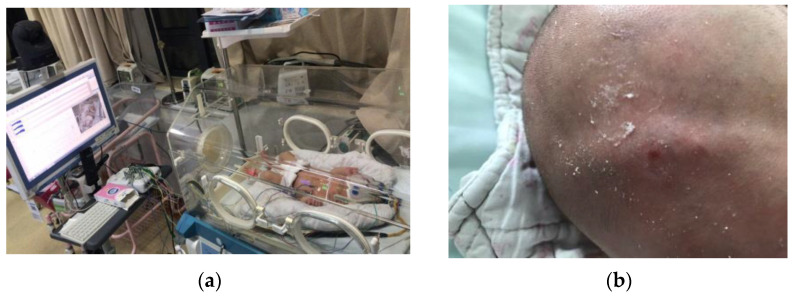
(**a**) Image of EEG acquisition setup. (**b**) Pressure sores on an infant.

Video electroencephalogram (VEEG), as the current gold standard for neonatal seizure detection, is widely used in clinical practice. Neonatal seizure is diagnosed through the acquisition and analysis of electroencephalogram (EEG) and video action signals [5]. However, video electroencephalogram technology has several disadvantages that are mainly manifested in the following aspects: First, EEG signals are not continuously monitored over a long time, making it difficult to accurately judge the nature of suspicious movements (e.g., normal behavior and convulsion) of newborns without synchronous EEG monitoring. A good number of minor seizures, such as continuous eye opening, eye movement or short-term gaze and chewing or swallowing, are very similar to normal behaviors of newborns, resulting in missed convulsions. Second, there are usually many instruments and equipment in the neonatal intensive care unit that are used to monitor numerous physiological parameters. There is also the use of scalp indwelling needles. The quality and stability of EEG signals will therefore be disturbed, and the placement of electrodes will be limited. At the same time, the wires connecting the medical instruments may interfere with the infant’s movement. Figure 1a shows collected EEG records containing a large quantity of artifact interference and clinical over-estimation of neonatal seizures. Third, the gel patch electrode or disk electrode commonly used to monitor EEG may cause skin allergies or even tears. In addition, prolonged contact with the electrodes may lead to pressure sores on the skin of newborns, as shown in Figure 1b. Negative stimulation such as pain and mental strain for newborns, which is not conducive to the treatment of convulsions, may also occur. Fourth, video electroencephalogram technology is time- and labor-consuming for medical personnel. In clinical practice, the analysis of neonatal seizures based on video EEG is mainly performed manually by experienced doctors who usually need to judge neonatal seizures by combining EEG signals and observing video information for several hours [5]. This method is not only time-consuming but also makes it difficult for doctors to keep track of the neonate’s condition in real time. EEG technology that is easy to judge and operate is essential for neonatal clinicians. Therefore, infant seizure automatic detection technology is an important area of research in seizure monitoring.

In addition to abnormal EEG signals, a seizure is usually manifested by behavioral and physiological signal changes, such as repeated movements of the arms, hands, legs and eyes and is sometimes accompanied by muscle contraction at a fluctuant velocity in the opposite direction as well as changes in the patterns of electrocardiogram (ECG) and respiration [1,6,7]. With the rapid development of related technologies in Body Sensor Networks (BSNs) over the past decade [8,9,10], researchers are searching for new automatic monitoring methods that differ from video EEG methods for monitoring seizures in children [11,12]. Monitoring seizures from a variety of electrophysiological and behavioral signals obtained using diverse modern signal processing methods, including electrocardiogram, myoelectricity, electrodermal activity and respiratory and behavioral signals, has been studied [13,14,15,16,17,18,19]. However, these studies have different shortcomings. For example, (a) only specific types of neonatal seizures with obvious behavioral abnormalities at the time of the attack can be monitored, (b) the false positive rate is higher and (c) they are only used to judge adult seizures, etc.

Current research has reported good detection results of neonatal seizures based on EEG signals. Tanveer et al. used a deep learning-based approach for neonatal seizure classification using EEG signals. The architecture they used is a two-dimensional (2D) convolutional neural network (CNN) that classifies seizure/non-seizure states. The dataset is annotated by three experts, thus three separate models are trained on individual annotations. After training/testing three individual models, the final model is composed of these three models. The average ACC and AUC given by the final model are 96.3% and 99.3%, respectively [20]. Tapani et al. develop a method for detecting neonatal seizures by adapting estimates of the correlation both in time (spike correlation, SC) and time-frequency domain (time-frequency correlation, TFC) based on the EEG. A total of 21 features defined in time, frequency and information theory domains and characteristics of autocorrelation analysis were extracted from the EEG signal. These features were fed into SVM classifiers. The proposed method was highly discriminative for neonatal seizure detection and gave AUC = 92%, SEN = 76% and SPE = 99% [21]. In addition, some studies have also presented a neonatal seizure detection system based on ECG, for example [22]. Lorenzo et al. presented an ECG-based neonatal seizure detector (NSD). The NRD uses a Generalized Linear Model (GLM), with features extracted from Heart Rate Variability (HRV) measures as input. The results of the GLM model as an ECG-based NSD gave ACC = 68%, SEN = 43%, SPE = 77% and AUC = 69%.

In this context, we proposed to solve the problems associated with neonatal seizure monitoring by combining “flexible sensor network” and “multi-modal signal fusion technology”. We diagnosed neonatal seizures by combing multi-modal physiological parameters (electrocardiogram, respiration) and motor signals to achieve comfortable, continuous and effective detection and prediction of neonatal seizures, hoping to provide new research directions and perspectives for neonatal seizure monitoring.

In our previous work [23], we developed a wearable multi-sensor platform for newborns to collect physiological and movement signals. In this paper, we further expanded on our previous work to detect neonatal seizures, designed a second-generation multi-sensor platform and developed an algorithm for automatically detecting neonatal seizures based on ECG, respiration and acceleration. Data for 38 neonates were recorded at the Children’s Hospital of Fudan University in Shanghai. The total recording time was approximately 300 h. Four of the patients had seizures during data collection. The total recording time for the four patients was approximately 34 h, with 30 seizures recorded. These data were evaluated by the algorithm. To evaluate the effectiveness of combining ECG, respiration and movement, we compared the performance of three types of seizure detectors. The first detector included features from ECG, respiration and acceleration records; the second detector incorporated features based on respiratory movement from respiration and acceleration records; and the third detector used only ECG-based features from ECG records. Our study illustrated that, compared with the detector utilizing individual modal features, multi-modal features could achieve favorable overall performance, reduced false alarm rates and higher F-measures.

Some of the experiments described in this paper have been described in a Ph.D. thesis [24].

## 2. Methods

**Figure 2:**
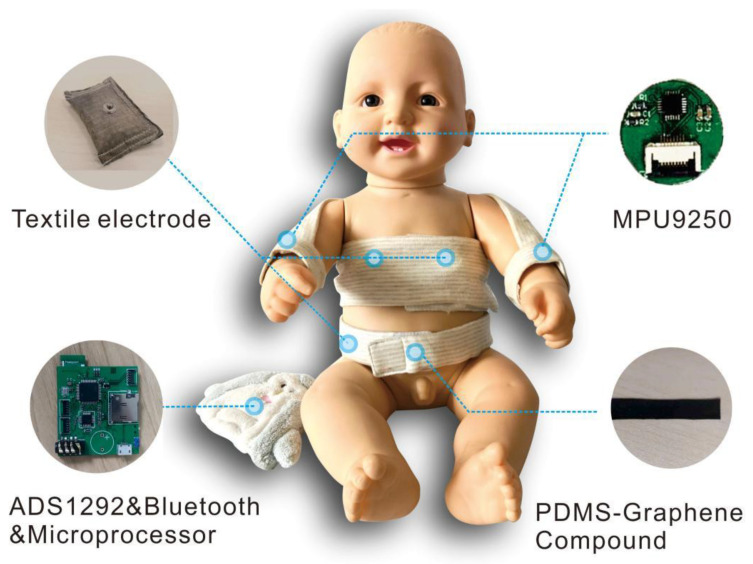
Prototype of the smart vest.

Based on previous research on wearable neonatal vital monitoring systems [10], we designed a wearable multi-sensor platform (MSP) for neonatal seizure monitoring, as shown in Figure 2. Compared with the design of first-generation smart clothing, the whole structure of second-generation smart clothing adopts an open front-end design to expose more skin during continuous monitoring and reduce the impact of clothing on newborns. The bandage design with a magic sticker is conducive to the fixation of sensors, which can make it more convenient for clinical nurses to operate. To ensure the comfort of the newborns, we integrated the sensors into the clothing to reduce interference of the components with the newborns. The garment side on which the signal processing module, the signal transmission module and the battery are placed is connected to a doll.

PDMS-Graphene was used to collect respiratory signals, textile electrodes were used to collect electrocardiogram signals and IMU was used to collect motion signals. All the signal acquisition modules were controlled and managed by the Micro Controller Units (MCU) at a sample rate of 250 Hz. All signals were sent to the terminal through Bluetooth. The data rate in the communication process between the Bluetooth and the terminal was 921,600 bits per second.

We previously carried out a systematic verification of the platform [24], including determination of the electrical properties of the new sensing materials, signal quality evaluation and comparison with the gold standard to verify the feasibility of the system. Verification experiments proved that: high-quality ECG signals can be obtained through the proposed textile electrode materials, with performance equivalent to that of commercial AgCl adhesive electrodes; accurate respiration data can be obtained through the stretching sensor based on the new PDMS-Graphene compound; and wrist movement signal can be obtained using the IMU sensor (MPU9250).

To verify the clinical feasibility of the multi-sensor platform for neonatal seizure detection, we obtained permission from the Research Ethics Committee of the Children’s Hospital of Fudan University [approval No. (2017) 89] to recruit infant patients for neonatal seizure detection. The neonatal seizure detection process included three parts: data collection, data analysis strategy and performance evaluation.

### 2.1. Clinical Data Collection

The VEEG acquisition system (niocolet) in the hospital and the multi-sensor platform (MSP) were used for simultaneous data acquisition. EEG signal and video information of the neonate were collected by the VEEG acquisition system as the gold standard for the detection of neonatal seizures, while ECG signals, respiratory signals and motion signals were collected by the proposed multi-modal wearable sensor system (MSP).

The patient recruitment criteria, including inclusion criteria, exclusion criteria and termination criteria are detailed as follows:

Inclusion criteria: (1) Age less than 60 days; (2) Patients with seizures in the clinic; (3) No anticonvulsant treatment before admission;

Exclusion criteria: (1) Patients with a history of respiratory disorder; (2) History of allergy to medical-grade skin adhesive or latex. (3) Patients with life-threatening diseases, such as shock, cerebral infarction and severe congenital malformations.

Termination criteria: (1) Condition of the subject continues to deteriorate during the study period, with even dangerous events occurring; (2) Inappropriate changes in the subject’s comorbidities, complications or special psychology; (3) Subjects experiencing adverse events.

**Figure 3:**
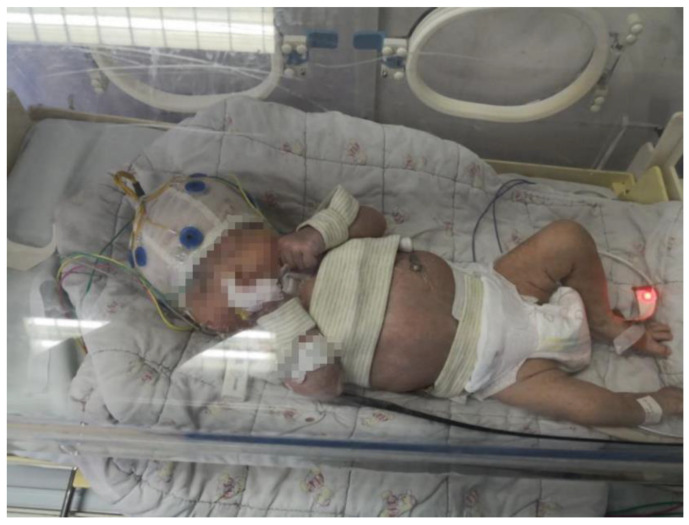
The environment of neonatal seizure data collection.

Long-term monitoring is required due to the sudden occurrence of neonatal seizures. Wearing the EEG cap of the EEG collection system for long-term monitoring will damage the neonate’s skin, therefore data can only be collected over a period of 4 h at any one time. The interval between the first and second data acquisition experiments on the same subject is 24 h. The experimental setup for data acquisition is shown in Figure 3. The EEG cap was placed on the neonate’s head to collect EEG signals. A camera was placed on top of the incubator to collect video information. The neonate was dressed in the smart vest, with the flexible electrode on the chest to collect ECG signals, the IMU on the wrist to collect body motion signals and the respiration sensor on the abdomen to collect respiration signals. The sampling frequency of the EEG acquisition system was 500 Hz and that of the multi-sensor platform was 250 Hz. During the experiment, data received by the multi-sensor platform were stored on a laptop and processed on a desktop using MATLAB R2019a (MathWorks Inc., Natick, MA, USA). EEG signals and video information were sent to the data storage center of the hospital and analyzed by medical experts to determine the occurrence of neonatal seizures. The following measurements were obtained by the principal investigator during each trial:

1. Subject’s age and gender.
2. Start time and end time of neonatal seizures. The time of seizure occurrence was determined by hospital experts through video and EEG analysis.
3. Attending NICU nurse rating of ease of use of the device, based on an 11-point Verbal Rating Scale (VRS) (0 = least difficulty of use, 10 = highest difficulty of use).

The following measurements were recorded electronically:

- ECG signal, respiration signal and motion signal collected by the MSP prototype and recorded on a Dell Laptop.
- EEG signal and video collected by the EEG acquisition system and record on a local data platform at the hospital.

A total of 38 patients (23 males and 15 females) who had experienced seizures at the hospital and did not violate the exclusion criteria were enrolled in the trial. The average age of the subjects was 36.8 days (median 37 standard deviation 8.3, range 20–57). Since intervals between multiple recordings for individual subjects exceeded 24 h, the average age of the subjects was calculated based on the time of each record. The average time of one experiment was 228.8 min (median 240, standard deviation 36.8, range 78–292). No patients suffered adverse respiratory events or skin irritation during the trial. In terms of comfort, 12 nursing staff (100%) rated the multi-sensor platform as 10 on VRS for ease of use (only for devices).

During the test, a total of 30 seizures were recorded in four patients. Seizures were not detected in most of the patients for several reasons. For example, the early diagnosis was wrong and the patient had no brain nerve damage, thus the EEG detected was normal. The detection time may also have been limited, with no seizure occurring within the 4 h window. Seizures were identified by an experienced neurologist reviewing video and EEG recordings and annotating the onset and offset of the manifestation of the seizures. Table 1 shows a broad overview of the patients who experienced seizures.

### 2.2. Data Analysis Strategy

**Figure 4:**
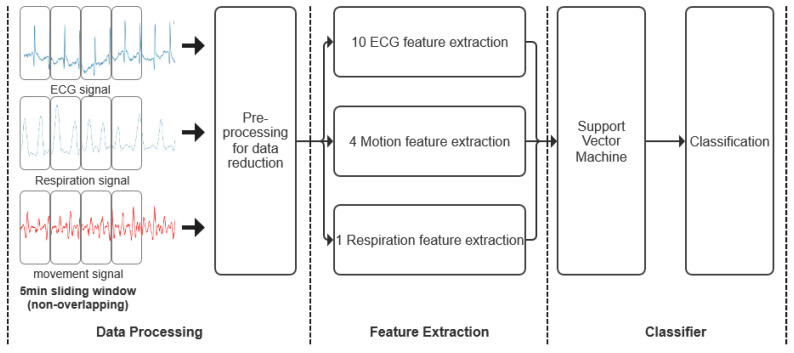
Overview of neonatal seizure detection architecture.

The various stages of neonatal seizure detection are depicted in Figure 4. A sliding window was used to extract 5-min epochs from both ECG, acceleration and respiratory recordings without overlapping. The data were then preprocessed to remove artifacts and reduce data for further processing. A total of 15 features, including mean R-R wave, respiration rate and total power features were extracted from the remaining epochs of ECG, acceleration and respiratory signals to form feature vectors. Finally, each feature vector was assigned to a seizure or non-seizure class using Support Vector Machine (SVM).

#### 2.2.1. Data Processing

Data from Table 1 were used to develop the classification algorithms. The analysis included complete records regardless of record length, record quality or the child’s awake or asleep state. A 5-min long non-overlapping sliding window was applied to ECG, acceleration and respiratory records to divide the data into epochs. The data were then preprocessed to remove artifacts and reduce the data for further processing. The details are as follows:

a. For ECG records, ECG is processed for R wave detection and HRV spectrum analysis. A large number of interfering signals can affect ECG signals, including 50 Hz power line interference, EMG signal interferences and baseline wandering. Therefore, at the preprocessing stage, these interfering noises are eliminated using a 5–15 Hz band pass filter. Next, to detect ECG R waves, the Hamilton and Tompkins algorithm was employed [25,26] to construct the RR-interval signal by measuring time intervals between consecutive R peaks. The heart rate variability spectrum was calculated by serial FFT (Fast Fourier Transform) using a Hanning window.
b. For respiratory records, Global linear drift was removed by subtracting the slope of the linear regression model of the data. Local signal drifts were corrected to continuous, minute-long sliding mean baseline windows, and padding was removed. Next, to extract the respiratory rate, the onset of inhales and exhales was estimated using the zero-crossing point detection algorithm. Based on the respiratory flow, the upward and downward zero-cross points were identified as the onset of inhales and exhales, respectively.
c. For movement records, acceleration data from each axis were low-pass filtered, with 47 Hz as the cut-off frequency, to remove the power line and high-frequency interference. After down-sampling acceleration signals by a factor of 2, a high-pass filter cutoff frequency of 0.2 Hz was applied to remove baseline drifts [27].
d. Seizures typically last 1–2 min, whereas patients are monitored continuously during hospitalization (several days). As such, there is a vast amount of non-seizure data (forming most categories), which causes a high imbalance in the data set. To reduce the computational workload and the degree of data imbalance during supervised learning, we labeled the 5-min signal after the onset of seizure as a seizure epoch, which contains seizure activities. The 25-min signal before the onset of the seizure and the 20-min signal after the offset of the seizure were labeled as non-seizure epochs. In cases where multiple seizures occurred close to each other within 5 min, the seizures were evaluated as a single seizure. Therefore, we adjusted our dataset to balance the number of seizure and non-seizure epochs. The ratio of seizure to non-seizure epochs for each patient was approximately 1:9. It is worth noting that where the interval between two seizures was greater than 5 min but less than 45 min, we collected an additional 20 min of normal data.

#### 2.2.2. Feature Extraction

A range of parameters has been used in the classification of neonatal seizures to describe ECG, respiration and movement modalities. Features extracted from ECG signals were obtained from the literature [28,29,30]. Features for acceleration-based seizure detection [17] and respiration activity recognition [31] were also added to the list, as shown in Table 2.

ECG feature:

A total of 10 features were computed to characterize each epoch and describe the time and frequency characteristics of ECG signals. There are ranges of mean RR, SDNN, SDSD, PNN50, SD1, SD2, CCM (Complex Correlation Measure), LF power, HF power and RR-interval signals.

Respiratory feature:

Breathing rate was calculated as the reciprocal of the average time between inhale onsets.

Movement feature:

The major energy band of daily activities falls between 0.3 and 3.5 Hz, whereas during seizures, the energy is typically concentrated at a frequency above 2 Hz [17]. To capture the spectral information of net acceleration, we computed the power spectral density using Welch’s method. Integrated powers within the 0–2 Hz and 2–5 Hz spectral bands were included as features. The total power within each epoch was also included as a feature. Moreover, the zero-crossing rate of the net acceleration was included for classification.

To summarize, a total of 15 features were computed from each epoch constituting the feature vectors, including 10 ECG features, 4 acceleration features and 1 respiratory feature. Each feature vector was assigned to a seizure or non-seizure class using Support Vector Machine (SVM).

#### 2.2.3. Classifier

Support Vector Machines (SVMs) are state-of-the-art binary classification methods that usually exhibit favorable resistance to overfitting and excellent performance in complicated pattern recognition problems [32,33,34]. An SVM can learn to separate the hyperplane from the decision boundary of two classes. This hyperplane is selected to maximize the classification margin, which is the geometric distance between the hyperplane and the boundary cases of each class (i.e., the support vectors) [35,36]. Moreover, SVMs can map the original finite-dimensional feature space into a much higher dimensional space by using a kernel function to improve the separability of the data. Gaussian Radial Basis kernel function (RBF) was selected as it provides non-linear mapping from the original feature vectors to a higher dimensional space. SVM is a good option for seizure detection tasks because its unique learning mechanism allows it to perform well on moderately imbalanced data without any modification [37]. Since SVM only takes into account those instances close to the boundary when building its model, the SVM is unaffected by negative instances far from the boundary—even if the number is large—which is important since the number of non-seizure instances far outnumber the number of seizure instances.

### 2.3. Performance Evaluation

We implemented a patient-independent seizure detection algorithm that excludes all data from the test patient in the training phase (leave-one-patient-out cross-validation). To allow the SVM to learn from previous examples of seizures in the test patient, we implemented double leave-one-seizure-out cross-validation when a patient had multiple seizure records available. The data length for each patient is shown in Table 3.

Generally, the confusion matrix is used to represent the decision made by the classifier. The matrix is composed of four data types: true positive (TP), false positive (FP), true negative (TN) and false negative (FN). True positive (TP) represents the correctly labeled period of seizure; False positive (FP) represents the incorrectly labeled period of seizure; True negative (TN) represents the correctly labeled non-seizure period; False negative (FN) represents the incorrectly labeled non-seizure period [38]. The performances of the developed seizure detectors were characterized based on their sensitivity, specificity, accuracy, false alarm rate per hour (FAH), F_1_-measure and AUC (Area Under the Curve). Sensitivity (SEN) and specificity (SPE) show the percentages of test seizures and test non-seizures identified by the algorithm, respectively [28].

(1) $$\mathrm{SEN}=\frac{\mathrm{TP}}{\mathrm{TP}+\mathrm{FN}}\;$$

(2) $$\mathrm{SPE}=\frac{\mathrm{TP}}{\mathrm{TN}+\mathrm{FP}}$$

Accuracy (ACC) gives the overall classification accuracy [39].

(3) $$\mathrm{ACC}=\frac{\left(\mathrm{TP}+\mathrm{TN}\right)}{\left(\mathrm{TP}++\mathrm{TN}+\mathrm{FP}+\mathrm{FN}\right)}$$

False alarm rate refers to the number of times in an hour that the system declares the onset of seizure activity without an actual seizure. However, when learning imbalanced data, the overall classification accuracy is not an appropriate performance measure, since a trivial classifier that predicts each instance as a majority class (non-seizure) would achieve very high accuracy but be of little use. As such, F_1_-measure and AUC were used to evaluate the performance of the SVM [40]. AUC is the area under the operating characteristic (ROC) curve. This is an important parameter for comparing the performances of different systems. The ROC curve is obtained by plotting the relationship between SEN and SPE.

(4) $$F=\frac{2\times P\times R}{P+R}$$

P denotes the precision and R denotes the recall.

(5) $$P=\frac{\mathrm{TP}}{\mathrm{TP}+\mathrm{FP}}$$

(6) $$R=\frac{\mathrm{TP}}{\mathrm{TP}+\mathrm{FN}}$$

To evaluate the utility of combining ECG, respiration and movement, we compared the performance of three types of seizure detectors. The first detector included features from ECG, respiration and acceleration records; the second detector incorporated respiratory motion-based features from respiration and acceleration records; and the third detector only used ECG-based features from ECG records.

## 3. Results

We reported the results of the evaluation of the neonatal seizure detection algorithm in the ECG-based mode, respiratory-acceleration-based mode, and aggregated (full) mode (Table 4). Assessment indicators included sensitivity, specificity, accuracy, false alarm rate per hour, F_1_-measure and AUC.

**Figure 5:**
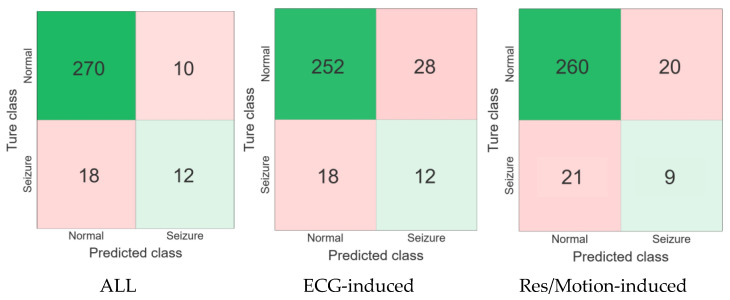
Confusion matrices for all models.

**Figure 6:**
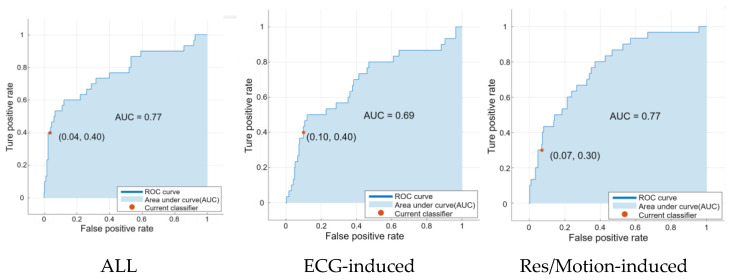
Receiver operating characteristic (ROC) curve analysis of data from the three seizure detectors.

To visualize the performance of the three neonatal seizure detection modes tested using data from four seizure patients, receiver Confusion matrix and operating characteristic (ROC) curve analyses were performed, as shown in Figure 5 and Figure 6. The ROC curves depict the trade-off between sensitivity (the percentage of recorded seizures identified by the detector) and false alarm rate as the decision threshold changes.

The results showed that the false alarm rate of the proposed algorithm in ECG-based mode was 1.29 times/h higher than in the respiratory-acceleration-based mode and multi-modal-based mode, while its accuracy, specificity and AUC values were 85.16%, 90% and 0.69 lower, respectively, than in the respiratory-acceleration-based mode and aggregated (full) mode. Moreover, the specificity, accuracy and F_1_-measure values of the aggregated (full) mode were higher than those of the ECG-based and respiratory-acceleration-based modes, reaching 96.43%, 90.97% and 0.461%, respectively. The false alarm rate of the algorithm was lowest under this mode, at only 0.46 times per hour. Overall, the results show that the seizure detection algorithm based on the aggregated (full) mode can improve performance.

## 4. Discussion

We proposed a smart vest as a multi-sensing platform embedded with non-invasive sensors based on flexible materials for neonatal seizure monitoring. We developed an automatic detection algorithm for neonatal seizures based on ECG, respiration and acceleration. The records of four patients experiencing seizures were evaluated by the algorithm. To evaluate the effectiveness of combining ECG, respiration and movement, we compared the performance of three types of seizure detectors. The first detector included features from ECG, respiration and acceleration records; the second detector incorporated features based on respiratory movement from respiration and acceleration records; and the third detector used only ECG-based features from ECG records. Our study illustrated that, compared with the detector utilizing single mode, multi-mode detectors could achieve favorable overall performance, reduced false alarm rates and higher F1-measures. The main reason for this phenomenon may be that different seizures have different signal features. Multi-mode platforms have better gain effects on machine learning compared with single-mode platforms. Different features can complement each other, thus the performance of a neonatal seizure detector model based on multi-modal signals is superior to models based on individual signals.

In addition, motor activity during seizures may affect the quality of ECG and respiratory signals, which will affect the machine-learning classification results. We will improve the quality of ECG and respiratory signals during motor activity by optimizing signal processing methods or optimizing sensors in future work. Furthermore, the duration of a neonatal seizure is less than 5 min, while the model we used was based on a 5-min window. This also means that the 5-min window of detection may contain one or more seizure events. Therefore, this model cannot detect the exact time of seizure occurrence. In the future, we will also attempt to develop a neonatal convulsion detection model based on multi-modal signals collected within shorter time windows in order to obtain as accurate a seizure event onset time as possible.

The results showed low sensitivity of the full-modal method, likely due to the small sample size resulting in under-representativeness. In the next step, we plan to adopt data augmentation to make up for the problem of small sample sizes. In addition, poor data quality, unknown seizure types (some are asymptomatic without abnormalities in respiration, electrocardiography and motor activity) and rebalancing approaches may lead to deviation and interpretation limitations. Furthermore, the current clinically used EEG monitoring equipment limits measurement time for each patient to 4 h due to considerations of patient comfort. In a future study, a more comfortable EEG device will be considered to prolong the measurement time, which could help us capture more seizure events.

It is worth noting that the feasibility of using non-EEG signals to detect neonatal seizures has previously been confirmed. For example, HRV analysis provides reliable biomarkers for brain damage in hypoxic ischemic encephalopathy (HIE) [8]. Malarvili et al. investigated the feasibility of using HRV time-frequency (TF) analysis for epilepsy detection [15]. The performance of seizure detection models based on non-EEG signals is often inferior to those based on EEG signals. The main reason may be that the current gold standard for diagnosing convulsions is based on EEG signals, and neonatal seizures caused by different etiologies may result in different non-EEG signal characteristics [2]. In other words, the currently proposed features of non-EEG signals often cannot cover all types of neonatal seizures. Therefore, improving the performance of multi-modal neonatal seizure detection platforms in the future may begin from the following two aspects: (1) Embedding wearable EEG signals into the platform. This approach is limited by the current wearable EEG electrode technology. (2) Exploring more features and trying to use deep neural networks to detect neonatal seizures. SVM is suitable for seizure detection when the number of features is small. In future work, we may try to improve system performance by increasing the number of features and using deep neural networks.

Additionally, in future work, we will further improve the structure and electronic properties of innovative materials to enhance the stability of the measured signal, particularly during movement. Furthermore, we will attempt to explore new EEG electrode materials to provide long-term EEG signal detection for multi-modal seizure detection platforms. Finally, we will conduct more clinical trials to verify the performance of wearable platforms for neonatal seizure detection. Data fusion and AI technology for processing clinical data will also be explored.

## Figures and Tables

**Table 1:** Overview of patients who experienced seizures.

No.	Sex	Average Age (Days)	Records	Record Duration per Time (min)	Seizure Events
1	M	36	1	236	1
2	M	41	1	132	1
3	F	30	6	249, 230, 232, 78, 191, 244	24
4	M	34	2	98, 231	4

**Table 2:** Overview of patients experiencing seizures.

Signal	Feature
ECG	Mean RR, SDNN, SDSD, PNN50, SD1, SD2 and CCM (Complex Correlation Measure), LF power, HF power and RR interval signals
Acceleration	Integrated power of 0–2 Hz, integrated power of 2–5 Hz, total power, zero-crossing rate
Respiration	Respiration rate

**Table 3:** Data length for each of the 4 newborns in the dataset.

No.	Data Length (min)	Seizure Events
1	50	1
2	50	1
3	1210	24
4	240	4

**Table 4:** Overview of the patients who experienced seizures.

Detector Based on	SEN	SPE	ACC	FAH	F_1_	AUC
ECG + acceleration + Respiration	40%	96.43%	90.97%	0.46 per hour	0.461538	0.77
ECG	40%	90.00%	85.16%	1.29 per hour	0.342857	0.69
acceleration + Respiration	30%	92.86%	86.77%	0.92 per hour	0.305085	0.77

## Data Availability

Data is unavailable due to privacy or ethical restrictions.
